# The Development of Diagnostic and Vaccine Strategies for Early Detection and Control of Human Brucellosis, Particularly in Endemic Areas

**DOI:** 10.3390/vaccines11030654

**Published:** 2023-03-14

**Authors:** Ayman Elbehiry, Musaad Aldubaib, Eman Marzouk, Adil Abalkhail, Abdulaziz M. Almuzaini, Mohammed Rawway, Ali Alghamdi, Abdullah Alqarni, Mohammed Aldawsari, Abdelmaged Draz

**Affiliations:** 1 Department of Public Health, College of Public Health and Health Informatics, Qassim University, Al Bukayriyah 52741, Saudi Arabia; 2 Department of Bacteriology, Mycology and Immunology, Faculty of Veterinary Medicine, University of Sadat City, Sadat City 32511, Egypt; 3 Department of Veterinary Medicine, College of Agriculture and Veterinary Medicine, Qassim University, Buraydah 52571, Saudi Arabia; 4 Biology Department, College of Science, Jouf University, Sakaka 42421, Saudi Arabia; 5 Botany and Microbiology Department, Faculty of Science, Al-Azhar University, Assiut 71524, Egypt; 6 Department of Optometry, King Fahad Armed Hospital, Jeddah 23311, Saudi Arabia; 7 Department of Family Medicine, King Fahad Armed Hospital, Jeddah 23311, Saudi Arabia; 8 Department of Medical services, Ministry of Defense, Riyadh 12426, Saudi Arabia

**Keywords:** zoonotic brucellosis, history, diagnostic approaches, vaccine development, public health

## Abstract

Brucellosis is considered one of the most serious zoonotic diseases worldwide. This disease affects both human and animal health, in addition to being one of the most widespread zoonotic illnesses in the Middle East and Northern Africa. Human brucellosis generally presents in a diverse and non-specific manner, making laboratory confirmation of the diagnosis critical to the patient’s recovery. A coordinated strategy for diagnosing and controlling brucellosis throughout the Middle East is required, as this disease cannot be known to occur without reliable microbiological, molecular, and epidemiological evidence. Consequently, the current review focuses on the current and emerging microbiological diagnostic tools for the early detection and control of human brucellosis. Laboratory assays such as culturing, serology, and molecular analysis can frequently be used to diagnose brucellosis. Although serological markers and nucleic acid amplification techniques are extremely sensitive, and extensive experience has been gained with these techniques in the laboratory diagnosis of brucellosis, a culture is still considered to be the “gold standard” due to the importance of this aspect of public health and clinical care. In endemic regions, however, serological tests remain the primary method of diagnosis due to their low cost, user-friendliness, and strong ability to provide a negative prediction, so they are commonly used. A nucleic acid amplification assay, which is highly sensitive, specific, and safe, is capable of enabling rapid disease diagnosis. Patients who have reportedly fully healed may continue to have positive molecular test results for a long time. Therefore, cultures and serological methods will continue to be the main tools for diagnosing and following up on human brucellosis for as long as no commercial tests or studies demonstrate adequate interlaboratory reproducibility. As there is no approved vaccine that prevents human brucellosis, vaccination-based control of animal brucellosis has become an important part of the management of human brucellosis. Over the past few decades, several studies have been conducted to develop *Brucella* vaccines, but the problem of controlling brucellosis in both humans and animals remains challenging. Therefore, this review also aims to present an updated overview of the different types of brucellosis vaccines that are currently available.

## 1. Introduction

Zoonotic brucellosis causes multi-systemic disease in humans and animals throughout the world; thus, the link between animal reproduction and human cases causes significant financial losses in many human living systems [1,2,3]. Each year, it is estimated that there are approximately 500,000 new cases of humans infected with brucellosis [4,5,6]. Among the cases recorded, the majority of cases occur in the Mideast, Central Asia, Adriatic nations, India, Central and South America, and Mexico [7,8,9]. Some countries do not have comprehensive case records or the infrastructure to report fevers of uncertain origin [5].

David Bruce discovered an organism called “*Micrococcus melitensis*” from autopsy samples taken from the spleens, livers, and kidneys of infected British soldiers in Malta. The organism was first identified by him as the cause of brucellosis, which was later renamed “*Brucella melitensis*” in his honor by Meyer and Shaw [10]. Hence, it is commonly known as brucellosis illness. Wildlife and farmed animals such as cattle, sheep, goats, camels, and pigs are affected [11]. It is also referred to as Bang’s disease, enzootic abortion, and contagious abortion in animals [12]. Other names include Malta fever, Mediterranean fever, intermittent fever, and undulant fever in humans. Professional techniques, including herd management, have established a risk group, but the disease is widely transmitted into the public due to consumption of infected milk and dairy products. Both breeders and the common public noticed a cluster of phenomena following the spread of the disease. There is significant diversity of zoonoses around the world, but the World Health Organization (WHO) has selected this disease as one of the most serious “ignored zoonoses” in the world, since it has an especially substantial impact on poverty-stricken nations [13]. *Brucella* has been designated as a hazard group III pathogen by the WHO due to its ease of aerosol transmission [14].

*Brucella* is a genus of Gram-negative bacteria [15,16] commonly referred to as coccobacilli, and is characterized by being small, non-capsulated, non-motile [17], and intracellular. Classical “core” *Brucella* species that can cause disease in humans include *Brucella abortus* (*B. abortus*), *Brucella melitensis* (*B. melitensis*), *Brucella suis* (*B. suis*) biovars 1 and 3, and, to a lesser extent, *Brucella canis* (*B. canis*), all of which are pathogenic. A number of new *Brucella* species have recently been reported in foreign frogs, and are genetically very variable in comparison to recognized classical *Brucella* species [18]. In 2009, Scholz and his colleagues, through the work they were conducting in Lower Austria, *Brucella microti* (*B. microti*) was isolated from the mandibular lymph nodes of a red fox and validly published as a new species based upon molecular analysis. It has also been demonstrated that *Brucella ceti* (*B. ceti*) was isolated from wildlife dolphins living in the Pacific Ocean and the Mediterranean Sea [19]. This shows that *B. ceti* is prevalent in marine environments and suggests that it has the potential to spread through aquatic animals, including dolphins, as well as other species. Furthermore, a previous report of a severe central nervous system infection caused by a marine mammal *Brucella* strain also supports the possibility that these microorganisms can spread from their original hosts and become infectious in people who live in a communal setting [20]. It was only recently discovered that a new species, known as *B. pseudogrignonensis,* was found in the blood sample of a man [21]. There is also a close relationship between atypical *Brucella* and core *Brucella*, which can be found in a similar ecosystem. According to Leclercq et al. [22] and Ryan and Pembroke [23], *Brucella* is one of several species within *Ochrobactrum*, but its exact place in the genus *Ochrobactrum* is still in question.

The prevalence of brucellosis in livestock, especially in low- and middle-income countries, has significant socioeconomic consequences [13,24,25]. Among the primary consequences of this disease are infertility, fetal death, late-gestation abortion, and decreased cattle productivity [26,27]. It is becoming more and more evident that brucellosis poses a serious public health risk in many underdeveloped countries due to the rise in incidences of morbidity in both humans and animals [28,29]. It is possible for humans to become infected with the disease either by coming into direct contact with sick animals or by consuming contaminated milk and milk products [29]. Brucellosis can also cause flu-like symptoms in humans (i.e., malaria, typhoid, streptococcal infections, and rheumatoid arthritis). Clinically, it cannot be distinguished from other influenza-like diseases, except rheumatoid arthritis [30]. As a result, physicians have encountered difficulties when dealing with acute human brucellosis [31].

In the diagnosis of human brucellosis, the microbiology laboratory plays an important role. Because human clinical symptoms are inconsistent and vague, it is very difficult to devise a proper therapy for these cases without using the microbiology laboratory [32]. In order to be able to correctly diagnose, there are three principles and methods that can be used in the laboratory: direct diagnosis through culture, indirect diagnosis through serological tests, and quick diagnosis utilizing PCR-based techniques. Among the many methods that are used to diagnose *Brucella*, clinical examination, bacterial culture from various biological sources, microscopy, biochemical tests, and serology are all parts of the standard diagnosis systems (such as the Rose Bengal test, latex agglutination test, complement fixation test, and enzyme-linked immunosorbent assay) [33,34,35]. It is important to note that these procedures require extensive time and resources to be performed, and some have limited sensitivity and/or specificity or require employees to have laboratory training and a biosafety level 3 facility [36,37]. In order to manage livestock brucellosis, there is no need to prescribe medication, since all animal species that test positive for the disease must be slaughtered [6,38]. Nevertheless, a latency may persist and hamper immediate success in disease eradication. 

The primary risk factors for occupational brucellosis include close contact with animal feces, veterinary services, and laboratories. These activities increase the chance of transmission through inhalation, ingestion, or direct contact with infected animals, animal products, or contaminated surfaces. People who work in these environments are at a higher risk for occupational brucellosis [39]. Handling potentially infectious animals, contaminated biological supplies, and live attenuated anti-brucellosis vaccines are all examples of risk factors for human brucellosis; however, more detailed knowledge regarding the specific risk factors for each occupation as well as how these risks can be measured remains to be elucidated [40]. To prevent the spread of brucellosis among professions that directly handle animals or animal products, effective preventive measures, such as the use of personal protective equipment, are necessary [40]. For more effective preventative measures to reduce the burden of the disease in groups exposed to their work activities, more precise data regarding the epidemiology of job-related brucellosis are necessary [40]. Knowing which industries and occupations are more likely to be exposed to brucellosis, as well as the factors that might increase their risk, would help to inform better prevention strategies, such as better hygiene practices, vaccination programs, and improved working conditions [9]. There are currently approximately 3.5 billion people in the world who are at risk of contracting brucellosis. This disease has serious consequences for the general population and results in significant economic loss as a result of therapy and reduced productivity, which leads to substantial economic losses [41,42]. 

The aims of brucellosis treatment, as summarized by Al Dahouk and Nöckler [43] and Doganay and Aygen [44], are to reduce or eliminate the symptoms, minimize complications, and prevent recurrences of the infection. By treating brucellosis rapidly, efficiently, and with early antibiotics, effective eradication with successful curing will be achieved. Several *Brucella* outbreaks have been reported in animal populations in the past few years. Despite early interventions to prevent the disease from spreading, human cases have relapsed [45]. The cause for these exacerbations is still being investigated, but it is unclear whether they were caused by the emergence of acquired resistance or whether they were caused by confinement within affected regions—for example, the parenchymatous organs and bones [6]. Although numerous research efforts have been made to determine how to eradicate brucellosis infection globally, the intermittent nature of brucellosis is still a serious concern and remains unclear to researchers [46]. Since the beginning of the twentieth century, there have been a number of inquiries and research projects that have been devoted to developing brucellosis vaccines. The production of vaccines against brucellosis has involved the use of inactivated, live-attenuated, and rough-attenuated vaccines [47]. *Brucella* vaccines play an important role in the prophylactic control of the disease. However, from both historical and contemporary perspectives, these vaccines do not provide an ultimate solution, as development of a national policy is required first, and enforcement later. Novel vaccines have not been yet employed, but their goals are to overcome adverse results such as the existence of zoonotic strains, to differentiate infected from vaccinated animals (DIVA), and to cause abortions similarly to wild strains; therefore, a simple approach is insufficient.

## 2. An Overview of Brucellosis’ History and Current Status

The first time that brucellosis was named a “Mediterranean relapsing gastric fever” was in 1861, when surgical assistant Jeffery Allen Marston, based in Malta, presented a case of the disease at the Royal Academy of Medicine in the United Kingdom [48,49,50]. During a survey of malnourished British soldiers living in Malta in 1887, David Bruce discovered a microorganism he named *Micrococcus melitensis*. This is a Gram-negative organism that can reach a length of 3 μm, and it can be found alone or in pairs in different environments [51]. There was a report published in 1895 by Bernard Lauritz Frederik Bang stating that *Bacillus abortus* had been isolated from the fetus, placenta, and uterine secretions of cattle during infectious abortions in four different places [50,52,53,54]. Wright and Smith established, in 1897, that brucellosis is a zoonotic disease through serum agglutination assays after they discovered antibodies specific to *B. melitensis* in human and animal serum [50,55]. Maltese citizen Themistocles Zammit, together with a large number of scientific members of the Mediterranean fever commission led by Bruce, established that goat’s milk was one of the reservoirs of the organism causing this disease, and the goats were exterminated in November 1906 [56,57]. 

The prevalence of brucellosis varies from country to country, but the countries located in the Mediterranean region are the most likely to be affected by this disease. In countries with low incomes, this illness is associated with a high morbidity rate. There have been several industrialized nations that have successfully eliminated this disease from their livestock [50,57,58,59]. From the middle of the 1980s until the beginning of the 1990s, the growth of human disease in North African countries (in particular, Morocco and Algeria) suggested the termination of this epidemiological rush. Furthermore, the illness received increased attention after a major epidemic struck Ghardaïa in 1984, with 600 cases identified. Later, the disease was found in several states across several countries, including the cities of Tlemcen (1986) and Sétif (1989) in northwestern Algeria. This infection was thought to be caused primarily by the consumption of cheese, as mentioned by Benhabyles et al. [60]. There was an increase of approximately 12% in bovine brucellosis infection rates in Algeria between 1969 and 1976 as a result of serological identification of the disease [61]. The overall seropositivity in sheep and goats between 1986 and 1989 was 2.12%, compared to 12.0% in 1985–1988. In the same study, 43.5% and 42% of sheep and goat flocks were affected, respectively [62]. 

## 3. Prevalence of Human Brucellosis

The prevalence of human brucellosis remains a global public health challenge due to its major health implications (such as infertility and pregnancy losses) and economic impact, particularly in areas with frequent animal infections [62,63]. Occasionally, *Brucella* infection can cause disease in newborns [64]. In certain circumstances, breast milk may be considered a potential mode of transmission for *B. melitensis* biovar 1 [65]. The prevalence of brucellosis in humans is determined by the type of domestic host, species of infected herds, and their density, as documented by over 1000 articles published by the WHO over the past 70 years (accessed on 1 December 2021 at https://www.who.int), with many of them describing the prevalence of the disease worldwide [29]. Among Middle Eastern nations recently reported to have brucellosis are Syria, Yemen, Saudi Arabia, Turkey, and Iran [8,9]. Moreover, it remains an epidemic in both Africa and Asia [66]. In areas with limited access to healthcare and diagnostic services, brucellosis prevalence is often not reported due to the lack of passive reports from hospitals and diagnostic labs. Several high-income nations, however, have documented a low prevalence of human brucellosis as compared to places where it is prevalent. In 2017, 381 cases of brucellosis were confirmed in 28 European countries, representing a rate of 0.09 for every 100,000 people per year [67]. The WHO reported that 67.2% of confirmed cases were reported in Greece, Italy, and Spain, with Greece having the highest rate of occurrence. 

In 2015, a randomized trial carried out in Costa Rica’s Huetar Norte Region (170 inhabitants) found human brucellosis prevalence at 12.5% among rural at-risk people. A Ministry of Health report confirmed that 144 human brucellosis cases had been reported for the entire country (at the time having 4.5 million inhabitants) but there had been only eight reported for the same northern region [68]. Additionally, inconsistent numbers have also been found in Colombia and Mexico [69,70], as well as other countries [71] that provide a similar picture. China has seen dramatic changes in human brucellosis over the past seven decades, particularly in the 1980s, when socioeconomic development grew significantly [72,73]. The National Brucellosis Prevention and Control Plan was implemented in China from 2016 to 2020 to eradicate the disease in both animals and humans [74]. A single dose providing lifetime protection is a recommended approach; however, different policies such repeated vaccination are still being employed in endemic areas.

The erroneous results may also be the outcome of a lack of instruments within a particular field, such as diagnostic tests and long-term epidemiological interventions. Alternatively, there are situations where there are some misunderstandings about how to attain relevant numbers, how the various tests function in certain epidemiological conditions, and the strategies for acquiring them. In other cases, there may be inadequate knowledge regarding how relevant data are produced and how the numerous tests perform in various epidemiological situations. Throughout the years, the “500,000 cases of brucellosis” figure, as well as the “percentage of undercounted cases” figure, has been repeated in many publications in the form of sentences such as: “There are about half a million cases of brucellosis reported worldwide every year. However, because of the disease’s imprecise clinical manifestations, there is a possibility that this number could be 10 times greater.” [75].

## 4. Diagnostic Approaches of Zoonotic Brucellosis

**Figure 1:**
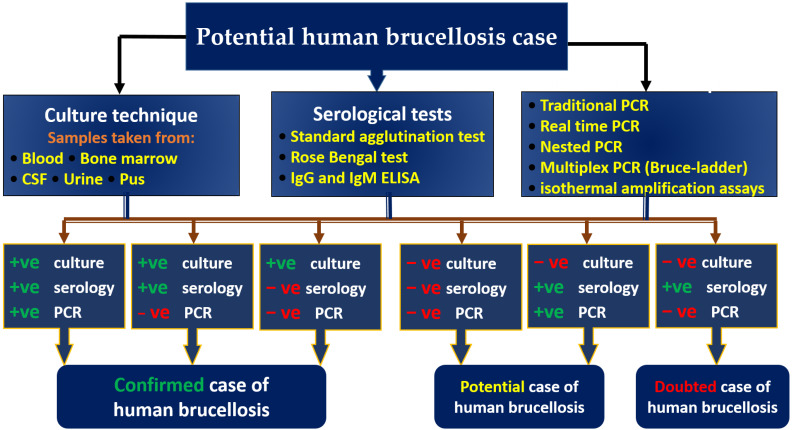
A diagnostic algorithm for zoonotic brucellosis that is based on laboratory findings.

A misdiagnosis of brucellosis may lead to unfavorable medication effects as well as missing more serious infections or non-infectious diseases that require immediate hospitalization [76]. An important factor that complicates *Brucella* infections is that they typically require continuous treatment with combinations of antibiotics that are generally not advised in the treatment of other contagious diseases [77]. In order to facilitate swift and effective clinical outcomes, it is crucial that this disease be identified in humans [76]. As shown in Figure 1, there are three different methodologies that can be used to identify human brucellosis microbiologically, including culture, serology, and molecular methods. The present study provides not only a thorough assessment of the relative advantages and disadvantages of these diagnostic techniques, but also a synopsis of the recent advancements, current status, and clinical applications of these diagnostic techniques.

### 4.1. Culturing Technique

An accurate diagnosis of human brucellosis requires the identification of the etiological agent from blood, bone marrow, or other tissues and bodily fluids. Various factors contribute to the rate of bacterial isolation, including the stage of the disease, the clinical specimen, previous antibiotic use, and culture technique [78]. The isolation of the *Brucella* species is a challenging task for many researchers, and many have attempted to overcome these challenges by using techniques that directly detect them in tissues or blood. However, none of these technologies has proven more reliable and effective than the isolation of bacteria itself [79]. In spite of the lack of ideal diagnostic sensitivity, *Brucella* cultivation is more likely to occur when known techniques are employed [76]. A combination of the traditional Ruiz-Castaeda biphasic technique and contemporary incubators is most effective in eliminating most of the biosafety problems associated with these pathogens. Both of these techniques detect bacterial growth without the danger of blind subcultures. When it comes to identifying brucellosis in clinical laboratories, culture is the gold standard. Since human brucellosis is always characterized by an initial bacteremic phase that precedes the development of its pathogenesis, peripheral blood cultures should always be performed as soon as cases of brucellosis are suspected [32]. Even though this method of diagnosis has a sensitivity between 10 and 90%, it is crucial for the accurate diagnosis of this disease [77]. It is possible to isolate *Brucella* species using blood cultures if serological tests are still negative or inconclusive [80,81]. 

Different blood culture methods have been used for the detection of *Brucella* species, such as the manual monophasic and biphasic methods [82], lysis-based blood cultures, blood clot cultures, and automated newly developed blood culture. Modern blood culture tools have improved *Brucella* species identification and improved the specificity of blood cultures [83]. The bloodstreams of patients with brucellosis initially exhibit a low bacterial burden, which cannot be revealed without following the suggested blood culture collection standards. To determine the severity of the infection, at least two or three different blood culture sets should be collected, rather than one [84]. The amount of bacteria circulating decreases as the illness worsens. In the course of the illness, the microorganism penetrates the phagocytes and multiplies, making isolation from the body difficult [85]. Standard culture practices need to be modified due to *Brucella* species’ delayed growth, low blood bacterial concentration, and carbon dioxide requirement [86]. Brucellosis of greater severity can be detected seven days after incubation, regardless of the ability of automated blood culture methods to allow five days [87]. The American Society for Microbiology and the WHO suggest that blood culture bottles be incubated for as long as one month, with blind subcultures in some cases. This protocol has many disadvantages, such as the cost and time commitment, structural difficulties, and delays in diagnostic testing [76].

*Brucella* organisms can spread outside the bloodstream within 24 h of primary hematogenous dissemination in 25–35% of patients. *Brucella* can also be isolated from other types of samples besides blood, such as bone marrow, urine, liver biopsy specimens, lymph nodes, and cerebrospinal fluid, by incubating samples in 5% CO_2_ at 35 °C for up to two weeks or inoculating specimens into broth media [88]. 

*Brucella* species and their natural hosts are closely related, so it is important to quickly identify *Brucella* cultures to prevent the spread of epidemics. Identifying *Brucella* is essential for preventing biological danger, verifying the presence of the disease in its infancy stages, distinguishing between wild and vaccine strains, and monitoring the source of infection. To determine the type of *Brucella* colonies, phage lysis and oxidative metabolism profiles, carbon dioxide requirement, hydrogen sulfide production, basic fuchsin (1:50,000 and 1:100,000 dilutions), and agglutination with monospecific A and M anticseras were used [89]. Considering the disadvantages of culture techniques, such as time requirements, hazards, and insensitivity, serology should be implemented to increase sensitivity.

A variety of non-phenotypic techniques can be applied to identify *Brucella* down to the species level, including matrix-assisted laser desorption ionization time-of-flight mass spectrometry (MALDI-TOF MS). However, MALDI-TOF MS has a variable level of accuracy, which may put it at a disadvantage versus phenotypic techniques [90,91]. As a precaution, it is advisable to use molecular techniques to confirm the identification of *Brucella* species [2]. In some clinical medical laboratories, broth from positive culture vials or colonies developing on a plate [92] is directly injected into the MALDI-TOF matrix to reduce the time required for analysis [93,94].

As a precaution against the possibility of exposure to live *Brucella* species, an intermediate step is often used to deactivate *Brucella* species by using 100% ethanol before regular protein extraction with formic acid and acetonitrile. This is an important step because it can reduce the possibility of exposure to *Brucella* species [2,95,96,97,98,99,100]. By utilizing MALDI-TOF technology, bacteria were identified at the genus and species levels. ATCC *Brucella* strains growing in synthetic blood cultures were correctly identified, and in some cases, their biovars were identified [101]. There is, however, evidence that other studies have shown that the Vitek MS system (bioMérieux, Craponne, France) based on the MALDI-TOF technology classified *B. melitensis* incorrectly as *O. anthropi* using accessible datasets [91]. According to Bruker Daltonics, Germany, the MALDI Biotyper purchased from that company showed inconsistent *Brucella* species differentiation, which proved that the observed protein patterns in the specimens were not sufficiently representative of the genomic evolution of *Brucella* [93,100]. A collection of 590 protein spectra was obtained from 84 *Brucella* isolates, including the most common species as well as exceptional strains, in order to build an updated Vitek MS library [90]. It was possible to distinguish clearly between *Brucella* and *Ochrobactrum* species and make identifications of the three main zoonotic species, including *B. aborus*, *B. melitensis*, and *B. suis,* due to the improved dataset. In order to independently confirm these encouraging results, a variety of wild-type isolates acquired from zoonotic and human sources with differing geographical histories must be evaluated. MALDI-TOF mass technology is advantageous in that the price per detected bacterium is low; however, this technology is expensive, which is why it is not widely available in most countries where brucellosis is prevalent [100].

Among the most common causes of laboratory-acquired infections are *Brucella* species, which have a multitude of biological characteristics that make it very easy for them to spread to lab workers, resulting in an epidemic of infection [102]. Lab workers are at risk when they handle specimens containing *Brucella* species [103,104] because processes or incidents can produce aerosols that can infect blood. Several portals of entry of *Brucellae* are relevant to laboratory work, such as the respiratory tract and conjunctival epithelium, as well as the gastrointestinal tract and skin that is abraded or exposed. The attack rate in clinical microbiology laboratories can vary from 10% to 100% depending on the inoculum used, the location of workers, and the exposure source [103,105,106]. Blood cultures taken early in the course of the illness may contain high concentrations of *Brucella* organisms. Misdiagnosis and injury to laboratory personnel can occur due to Gram stain misidentification [107]. The danger of exposure is increased in underdeveloped countries because of inadequate safety equipment [108]. According to the Turkish laboratory, 10 (18%) out of 55 workers were infected with the disease, and the estimated hazard was 8% (10/125) per employee per year [108]. Lab exposures and laboratory-acquired brucellosis cases persist despite laboratory safety measures and post-exposure guidelines [109]. Several effective exposure and infection prevention strategies are available to minimize laboratory exposures to *Brucella* species and prevent other laboratory-acquired diseases. Laboratories and doctors should consult each other when identifying specimens [110]. A precaution taken in laboratory settings is to keep unidentified specimens in a biological safety cabinet until a highly contagious infection is ruled out.

### 4.2. Serodiagnostic Approaches

Serological tests are prioritized in human diagnosis due to being robust. However, they were standardized as diagnostic tests against animal brucellosis especially in subclinical infections; thus, the same can be achieved in humans. A number of serological tests can be used to indirectly identify particular antibodies in patients’ serum. Basically, an interpretation criterion that has been established over a long period of time is whether or not an extremely specific titer can be obtained by an agglutination test, whether there is a cutoff value in an enzyme-linked immunosorbent assay (ELISA), or whether a distinct wavelength can be detected by a lateral flow immunoassay. As a consequence of lab setting variations as well as historical and epidemiological variables (such as aging, illness duration, workplace hazards, patient demographics, endemicity, and recurrence) [111,112], these parameters are frequently contentious. Serological tests have limitations, such as their limited sensitivity, difficulty in interpreting results, and inability to differentiate between the current infection and the previously acquired infection [43]. In brucellosis-endemic areas, false positives can result from cross-reactivity with pathogens that are not targeted by the test or from the discovery of immunoglobulin that is associated with a previously exposed infection [113], which can present a major obstacle for treatment. Despite these limitations, serological tests provide an inexpensive, simple, and highly accurate diagnostic tool for the detection of human brucellosis in areas of high endemicity and a low-to-middle income level.

For the detection of human brucellosis, there are numerous immunological assays available, with most of these being used for the detection of the disease in animals. The detection of non-agglutinating antibodies can be achieved through acidic pH tests (Rose Bengal test (RBT) or *Brucella*capt), Coombs tests, or immunoassays using *Brucella* lipopolysaccharides (LPS) and anti-IgG or anti-IgA conjugates [76]. The complexity and inherent features of antigenic structures, such as those found in cytosolic proteins, surface proteins, and immunodominant LPS, make serological tests significant diagnostic markers. For indirect fluorescent-antibody (IFA) assays, whole cell preparations are being utilized [35]. As a matter of fact, for the laboratory diagnosis of brucellosis, the majority of serological tests are classified into two kinds of tests: those that target the brucellar smooth LPS (S-LPS) and those that target its cytosolic proteins. Smooth *Brucella* species induce a strong humoral immune response that is primarily characterized by the production of antibodies to S-LPS [76]. In the first week after infection, IgM levels are elevated (which can be detected by agglutination tests such as RBT and slide agglutination test (SAT), followed by IgG1 levels in the second week, and, finally, IgG2 and IgA levels in the third week [76]. Due to the lack of the O-polysaccharides (OPS) antigen in *B. canis*, the risk of misdiagnosis of human infection may occur [114].

As a screening test for brucellosis caused by *B. abortus*, *B. melitensis*, and *B. suis* [113], the RBT, based on SAT, and a newly developed SAT miniaturized test [43,115], were used as the methodologies targeting S-LPS. In accordance with the recommendations made by numerous global institutions, the RBT should be used in combination with other methods, such as culture or other serology, for detecting human brucellosis [116,117]. It is widely believed and proven by a heavy body of literature that RBT can be used in a variety of circumstances [118,119]. Although RBT is a commonly used technique, there have been some challenges associated with it, such as misleading negative results caused by prozones and an inability to recognize non-agglutinating immunoglobulins (IgA/IgG) [43]. The RBT has demonstrated a high level of specificity in some studies; however, the application process of the RBT to healthcare settings has encountered a number of known obstacles. Its strong agglutinating activity makes IgM most effective in areas nearby hospitals. It is still possible for SAT to produce false-negative findings, even under these conditions, if the incubation period of the disease is prolonged [79]. Due to the predominance of non-agglutinating antibodies in long-evolution cases, the diagnostic performance of SAT is poor in areas with restricted access to medical facilities. The SAT with serum dilution, however, remains an effective diagnostic tool when necessary precautions are taken, as it overcomes the issues of blocking antibodies and prozone, is cost-effective, and is not overly complex. 

The fine chemical structure of the OPS of the LPS determines the serospecificity of the bacteria [120]. The application of serial dilutions to RBT with a 1:8 cut-off increases the specificity of the test without a significant loss of sensitivity, complexity, or time [113]. In addition to the detection of cytosolic proteins using ELISA, the same method has been successfully used for the detection of neuro-brucellosis in cerebrospinal fluid [121] and to identify immunoglobulins in different animal hosts and humans [122,123,124]. The ELISA assay previously demonstrated exceptional sensitivity (98.3%) and specificity (99.7%) for detection of human brucellosis [125]. High cost and infrastructure requirements make ELISA a secondary test in healthcare settings with low incomes [126]. As an initial screening method, it is possible to quickly detect brucellosis using a portable quantum dot immunochromatographic test strip. This is an easy and convenient method, and is available nowadays [127]. Unlike enzyme immunoassays and lateral flow immunochromatography, agglutination-based assays do not have the capability of distinguishing between different types of antibodies present, which makes them less accurate [32]. As a result of the achievements of increased sensitivity, rapid and accurate results, a reduction in costs, and easy implementation of these antigenic assays, experimental antigenic assays have been developed, such as synthetic oligosaccharides and recombinant *Brucella* proteins [128].

### 4.3. Genotypic Approaches

Genomic techniques can be utilized to detect brucellosis, both in humans and in animals, with great accuracy and speed. The capability of genotypic approaches to be successful for a considerable amount of time remains to be a significant advantage when clinical significance is ambiguous and individuals appear to be asymptomatic. There is, how-ever, the possibility that a positive result does not always mean that an infection is continuing. A significantly reduced bacterial inoculum may reflect a bacterial inoculum in routinely subjected normal individuals, DNA from dead organisms, or patients who have had adequate treatment for their ailments. Although serological tests and very sensitive nucleic acid amplification techniques have become increasingly common for the laboratory diagnosis of brucellosis, cultures remain the “gold standard” due to their clinical and epidemiological relevance [32]. Due to the specific nature of molecular approaches, findings from these approaches should be carefully interpreted, keeping in mind the relevant clinical and epidemiological contexts at all times. Molecular brucellosis testing has consistently shown that serum samples have a superior yield when used first on peripheral circulation with satisfactory completion, as they are an optimal sample for molecular analysis of human brucellosis [129,130]. Brucellosis may also be genetically characterized with samples obtained from the cardiac, urogenital, bone marrow, and peripheral nervous systems, which can aid in diagnosing focal brucellosis affecting any tissues or organs where civilizations are frequently negative [131,132]. The DNA extracted from formalin-fixed paraffin-embedded tissue was also tested in accordance with the approved DNA extraction procedures [133].

It has been reported that many molecular tests are used to identify genes that are responsible for making outer membrane proteins. A few of the more important genes are *OMP2* and *OMP31,* as well as the *omp28* gene, which is also called the *bp26* gene [134,135]. There are a few other gene targets that may be utilized for the molecular diagnosis of *Brucella* infection besides *16S rRNA* and the insertion sequence *IS711*, as cross-reactions have been detected in both genes. As a result of the fluctuation of *IS711* sequences and its absence in some strains, its performance has been questioned. In addition to being highly immunogenic, the most commonly selected gene, *bcsp31*, is also responsible for producing an immunogenic membrane protein [134,136,137]. In molecular techniques, there is a wide variety of amplification methodologies used to achieve amplification of DNA. Real-time PCR, quantitative RT-PCR, multiplex RT-PCR, nested PCR, in-house PCR, PCR-enzyme immunoassay in a microplate format, and traditional PCR procedures are some of the amplification methodology methods used [76,138]. Using a multiplex PCR assay, it is possible to develop a simple and easy one-step test for the detection of *Brucella* in a sample with significantly higher accuracy than traditional testing methods [139,140,141]. There are many advantages of this assay: in comparison with previously developed PCRs, the main one is its ability to distinguish between *Brucella* species and vaccine strains (S19, RB51, and Rev. 1) in a single step [142]. In addition, certain technologies have recently been developed for the detection of *Brucella* species, the most recent being a loop-mediated isothermal amplification (LAMP) method. Compared to PCR, the LAMP assay offers superior results due to its ease of use, ease of assembly, rapid response time, and visual recognition. Because of its simplicity and the low cost of the equipment used, including a laboratory water bath that maintains a stable temperature of 63 °C, it is well suited to the purpose of the test [143]. A LAMP test can be performed without the need for electrophoretic analysis, which is in contrast to PCR testing [144]. The LAMP assay is highly sensitive and specific, and can be completed in less than an hour [145]. It is also more cost-effective than alternative molecular techniques, since it does not require the use of expensive equipment or reagents. It can, therefore, be used in areas where resources are limited but the need for quick and accurate diagnosis is high.

Currently, there are very few competitive genetic methods for detecting brucellosis in both humans and animals, and there are hardly any comparative studies that have compared the effectiveness of commercial and homemade molecular methods. In addition, sometimes the results are based on very small sample sizes, making it difficult to find comparative studies [146,147]. 

By sequencing, it is possible to discover molecular mechanisms that explain variations of biotypes and evolutionary relationships for human brucellosis [148,149]. Identifying these targets could be a significant step forward in the development of diagnoses and vaccinations aimed at combating and treating brucellosis [150]. As part of the diagnosis process, it is necessary to use expensive, specialized equipment that is difficult to obtain. In low-income countries, the current availability of next-generation sequencing technologies is woefully insufficient to meet the requirements for research carried out in those countries. The fact that there is no specific test for definitively identifying a bacterium is the primary reason for the necessary integration of multiple techniques for *Brucella’*s diagnosis [149,151,152]. Although there are valid and reliable advertising and home-made molecular techniques that can ensure a high degree of accuracy and precision of results, conventional methods of culture and indirect methods of serology continue to be the primary methods of diagnosing brucellosis and following up on infections caused by *Brucella* species.

## 5. *Brucella* Vaccine Development

### 5.1. Availability of Human Vaccines

Vaccination has been well-established to be a crucial element in the control and prevention of brucellosis [153,154,155]. Currently, there is no human vaccine available for brucellosis, and the animal vaccines that are available have been deemed unsuitable for use by the general population due to concern regarding the possibility of causing serious illness [156]. Therefore, this has led to an increase in interest from researchers in developing a vaccine to prevent humans from contracting brucellosis. Despite the lack of a safe and effective *Brucella* vaccine for humans, animal vaccinations are essential not only to safeguard animal health, but also to prevent the spreading of diseases transmitted from animals to humans. Several drawbacks of these vaccines have been reported by Darbandi et al. [157]. Among these are: (i) they remain virulent in humans; (ii) they cause abortion in pregnant animals; (iii) it can be difficult to differentiate between infected and vaccinated animals due to the persistent serological response they induce; (iv) they are relatively unstable. However, a variety of vaccines have been developed so far to be used in humans, yet each of these vaccines has drawbacks that make their application difficult. In the case of the S19 *B. abortus* vaccine, for instance, which is administered subcutaneously by scarification, there is very limited immunity for a short period of time and it is very important to receive booster doses on a regular basis. An additional problem with this type of vaccine is its tendency to cause hypersensitivity reactivity. In general, *B. abortus* 84-C and M-104 have been found to be safe when administered intradermally or by spray, but this is not to say that they cannot cause serious reactions if improperly administered or if they are applied to susceptible individuals [153].

### 5.2. Live Attenuated Vaccines: Policies and Limitations

*Brucella* vaccines (usually live attenuated strains) play a critical role in combating brucellosis by endowing animals with immune protection and prevention of the spread of disease on farms. Most importantly, animal vaccination reduces abortion risks and thereby increases environmental safety. Currently, there are three different types of vaccines that are being distributed across the world against animal brucellosis: *B. abortus* S19 and *B. abortus* RB51, which are for cattle; and *B. melitensis* Rev. 1, which is for small ruminants [71]. Each of them is known to be 70% effective and has been administered efficiently; however, due to residual pathogenicity, which could cause illnesses to emerge, they are not suitable for use in humans [155]. The S19 vaccine is still the most cost-effective vaccine for the prevention of *B. abortus* infection in cattle at present. The RB51 strain of *B. abortus*, a spontaneous R-mutant, is used as a live vaccine, and is less effective (requires revaccination) and more expensive compared to other live vaccines. 

With the implementation of eradication programs, the use of the S19 and RB51 vaccines, as well as Rev. 1 in small ruminants, will become difficult because these vaccines produce antibodies that can be detected through serological tests [71,158]. These vaccines, however, are subject to some limitations that make their use difficult; for example, S19 and Rev. 1 may cause abortion in pregnant animals, and antibody titers resulting from vaccination may persist for a prolonged period in vaccinated animals [159,160]. Therefore, a safe vaccination approach, such as limiting animal age at vaccination and using a single full-dose lifetime protocol, will reduce the risks associated with persistent antibodies. Moreover, in some regions, repeated vaccination protocols at two-year intervals can be recommended in order to increase herd vaccination coverage.

*B. abortus* RB51 has been used since 2002 instead of *B. abortus* S19 for the vaccination of cattle [71,161]. The RB51 vaccine has more recently been widely distributed in sub-Saharan African nations [162]. Blasco [163] found that conjunctival vaccination with Rev. 1 effectively suppresses *B. melitensis* infection in sheep and goats, unlike rough RB51. Another study revealed that the RB51 vaccine is effective in controlling brucellosis in cattle regardless of the species [164]. 

One of the most promising strains of SR-*B. abortus,* which was discovered in Russia, is strain 82. In 1961, Salmakov discovered this strain through the selection of colonies derived from an aborted bovine fetus [165]. Vaccine strain 75/79-AB of *B. abortus* with marginal agglutinogenic properties was another strain that was used in the field regarding *B. abortus* vaccination in the Russian Federation. Its use was coordinated according to the results of serological testing within three months of immunization, and vaccination of pregnant heifers occurred regardless of gestational stage [166,167,168]. The *B. abortus* 104 M vaccine has a long history of being used in China for the prevention of brucellosis since 1965 [72]. As far as safety is concerned, this strain has been poorly defined, is hard to find, and is not readily accessible. In order to develop an effective and safe brucellosis vaccine for the general public, extensive research is necessary. There is a live attenuated *B. suis* vaccine known as the *B. suis* S2 that is the most widely used brucellosis vaccine in China [169].

Recently, there are numerous strategies that have been put forward to eradicate brucellosis. Among these are the limitation of animal translocation from diseased herds to healthy ones, the use of live vaccines such as S19 and Rev. 1, and a substantial preventative coverage, as well as the proper diagnostic methods. Vaccination has several advantages, such as preventing the spread of infection between humans and animals, reducing the incidence of brucellosis, reducing shedding rates among animals, and disrupting the ability to transmit diseases from one animal to another [170]. There may be some drawbacks to vaccination, including the development of field strains that are persistent or resistant to the immunity provided by the vaccination, the risk of unrecognized latency which may burst later on, and contribution to the false impression that infection has been beaten by vaccination [171]. 

### 5.3. Novel Vaccines with Recombinant Genes, Proteins and Vectors

The economic losses resulting from common illnesses between humans and animals [172,173] is motivating investigators to investigate vaccine candidates such as subunit vaccines [174], bacterial vector-based vaccines [175], and vaccinations utilizing overexpression of protective homologous antigens [173]. There is a wide range of genes that have recently been used, including L7/L12, BLS, BCSP31, SOD Cu/Zn, Omp16, P39, and BAB1-0278. It is generally not necessary to use adjuvants in DNA vaccinations [155]. It has been proven that the DNA vaccine for *B. abortus*, BAB1-0278, protects mice against infection. The effects of DNA vaccines containing BAB1 0273 and/or BAB1 0278 and SOD C on mice are low, although they induce immune responses [176]. A DNA vaccine candidate for p39 and/or groEL, together with other DNA-based vaccine candidates, needs to be repeated a number of times despite the fact that it provides only modest-level control. As a result, there is still much work to be accomplished in this field [177]. In order to improve the immune function of *Brucella* DNA vaccines and improve their effectiveness, it is recommended that cytokines be developed as adjuvants (SOD with IL-18 or IL-12), and a number of antigens could be incorporated into DNA vaccines (Omp16 and L7/L12) [155].

### 5.4. Expectations for Development Human Brucellosis Vaccines 

For the development of new *Brucella* vaccines, it is essential that the pathogenicity of the bacteria and the host be thoroughly analyzed. DNA vaccines are suitable for diseases that require cellular immunity. Human studies have shown that these vaccines induce weaker immune responses than mice; therefore, they need to be optimized [157]. Optimization, however, increases pro-inflammatory reactions [178]. New optimization methods can be used to improve delivery and codon optimization [179]. There are many methods that can be used to accelerate the development of a *Brucella* vaccine, including signature transposon mutagenesis with transposon tags, green fluorescent protein-expressing *Brucella* strains, and knockout mice. Using a broad genomic analysis and bacterial imaging both in vitro and in vivo, knockout mice and other genetically manipulated mice can be used to increase the likelihood of discovering weak strains as well as to speed up the pace of vaccine production [180]. It is important to consider many factors when developing *Brucella* vaccines: first, how to obtain authorization; second, evaluating the vaccine’s effectiveness in two experimental animals, a mouse and a monkey; and, finally, testing the vaccine for safety, immunogenicity, and effectiveness. A preventive measure should be expected to have an impact when it is impossible to test and demonstrate its efficacy on humans [156]. 

## 6. Conclusions

Although brucellosis is a rare infection that can be transmitted from person to person, it is a zoonotic infection that can be transmitted to humans from infected animals. There is a common view among the medical community that the main method of eradicating this disease in humans is through widespread vaccination of animals, along with testing and slaughtering. A culture remains the “gold standard” for brucellosis diagnosis even though serological markers and nucleic acid amplification techniques have been extensively used in the laboratory diagnosis of brucellosis. Despite this, serological tests are still commonly used in endemic regions because they are low-cost, user-friendly, and robust in predicting negative results. In addition, because of their high sensitivity and specificity, molecular approaches can be used as quick tests to detect brucellosis within a few hours. As there is no approved vaccine that prevents human brucellosis, vaccination-based control of animal brucellosis has become an important part of the management of human brucellosis. Generally, live-attenuated vaccines, commonly known as *B. abortus* strain S19 and *B. melitensis* strain Rev. 1, are one of the most popular ways to immunize animals around the world against brucellosis. Nonetheless, they have several disadvantages, including the induction of abortion in pregnant animals, pathogenicity for humans, the development of anti-*Brucella* antibodies that interfered with the serodiagnosis of brucellosis, and resistance to antibiotics used to treat brucellosis. Currently, there is no vaccine available against human brucellosis. In this regard, vaccination-based control of animal brucellosis has contributed significantly to human brucellosis management.

## Data Availability

Not applicable.
